# The E-Id Axis Instructs Adaptive Versus Innate Lineage Cell Fate Choice and Instructs Regulatory T Cell Differentiation

**DOI:** 10.3389/fimmu.2022.890056

**Published:** 2022-05-06

**Authors:** Reiko Hidaka, Kazuko Miyazaki, Masaki Miyazaki

**Affiliations:** Laboratory of Immunology, Institute for Life and Medical Sciences, Kyoto University, Kyoto, Japan

**Keywords:** T cell versus ILCs, Rag gene expression, E-Id axis, T-lineage commitment, Treg differentiation

## Abstract

Immune responses are primarily mediated by adaptive and innate immune cells. Adaptive immune cells, such as T and B cells, evoke antigen-specific responses through the recognition of specific antigens. This antigen-specific recognition relies on the V(D)J recombination of immunoglobulin (*Ig*) and T cell receptor (*TCR*) genes mediated by recombination-activating gene (*Rag*)1 and *Rag2* (*Rag1/2*). In addition, T and B cells employ cell type-specific developmental pathways during their activation processes, and the regulation of these processes is strictly regulated by the transcription factor network. Among these factors, members of the basic helix-loop-helix (bHLH) transcription factor mammalian E protein family, including E12, E47, E2-2, and HEB, orchestrate multiple adaptive immune cell development, while their antagonists, Id proteins (Id1-4), function as negative regulators. It is well established that a majority of T and B cell developmental trajectories are regulated by the transcriptional balance between E and Id proteins (the E-Id axis). E2A is critically required not only for B cell but also for T cell lineage commitment, whereas Id2 and Id3 enforce the maintenance of naïve T cells and naïve regulatory T (Treg) cells. Here, we review the current knowledge of E- and Id-protein function in T cell lineage commitment and Treg cell differentiation.

## Introduction

Innate immune cells and adaptive lymphocytes cooperatively evoke immune responses aimed at protecting our bodies from invasion of the pathogens. Innate immune cells, such as macrophages, neutrophils, and dendritic cells, are activated by pattern recognition receptors (PRRs) that recognize microbial components. On the other hand, adaptive lymphocyte T and B cells recognize specific antigens through diverse antigen receptors. This specific immune response relies on the V(D)J recombination of the immunoglobulin (*Ig*) and T cell receptor (*TCR*) genes mediated by the recombination-activating gene (*Rag1/2*). The assembly of the *TCR* and *Ig* genes from the arrays of variable (V), diversity (D), and joining (J) gene segments is initiated by a Rag1 and Rag2 protein complex, which recognizes and cleaves the recombination signal sequences (RSSs) flanking the V, D, and J segments of the *Ig* and *TCR* genes (1, 2). The expression of the *Rag1/2* genes is stringently controlled. These genes are expressed only in T and B progenitor/precursor cells, meaning that *Rag1/2* expression is a hallmark of the adaptive lymphocyte lineage.

Common lymphoid progenitors (CLPs) can give rise to T cells, B cells, innate lymphoid cells (ILCs) including natural killer (NK) cells, and dendritic cells (DCs). Once lymphoid progenitors from the fetal liver or bone marrow (BM) migrate into the thymus, they receive Notch1 receptor signaling through the interaction with Delta-like 4 (DL4)-expressing thymic epithelial cells and commit to the T cell lineage (3–6). After T cell lineage commitment, TCRβ and/or TCRγ/δ V(D)J gene recombination is initiated in immature CD4^–^CD8^–^ (double negative; DN) cells. DN cells are divided into multiple distinct stages distinguished by surface expression of CD44 and CD25 (DN1-4). In DN1 cells, early T cell progenitors (ETPs) are defined by CD25^–^CD44^+^KIT^hi^ expression, and committed T progenitor (pro-T) cells start expressing CD25 (DN2) since CD25 is a direct target of Notch signaling. Following the success of productive TCRβ recombination in DN3 cells (CD44^–^CD25^+^), DN3 cells start proliferating and differentiating into DN4 cells and further into CD4^+^CD8^+^ (double positive; DP) cells (T precursor (pre-T) cells). Recombination of the TCRγ/δ gene occurs concurrently with TCRβ recombination in DN2-3 cells (7). Upon reaching the DP stage, thymocytes exit the cell cycle (resting DP cells) and start TCRα VJ recombination (8, 9). DP cells that succeed in the production of a functional TCRα/β undergo positive and negative selection, which permits the developmental progression of T cells that have acquired a TCR with moderate affinity for self-antigens associated with major histocompatibility complex (MHC) class I (for CD8 single-positive (CD8SP) cells) or class II (for CD4SP cells) (10). The population of CD4SP cells that react more strongly with self-antigens associated with the MHC in the thymus differentiates into distinct regulatory T cells (Tregs), which specifically express the transcription factor (TF) Foxp3 and play an indispensable role in suppressing autoimmunity and excessive immune responses (11). On the other hand, innate type of T cells also arise from DP cells, which are selected by CD1 for invariant natural killer T (iNKT) cells and by MHC-related protein MR1 for mucosal-associated invariant T (MAIT) cells (12, 13). In these processes, sequential expression of an ensemble of TFs specifies the lineage-specific gene expression program and function through the regulation of the enhancer repertoire and activities (14, 15). However, the precise molecular mechanisms of how lineage-specific TFs synergistically regulate enhancer activities and how these factors cooperatively orchestrate the changes in chromatin architecture for appropriate gene expression remain unclear.

E proteins are basic helix-loop-helix (bHLH) TFs involved in multiple hematopoietic developmental processes, and mammalian E proteins include E12, E47 (from the *E2A*;*Tcf3* gene), E2-2 (*Tcf4*), and HEB (*Tcf12*). E proteins bind to the E-box motif (CANNTG) within the cis-regulatory element (CRE, enhancer region) of the target genes by forming homodimers or heterodimers. In contrast, Id proteins contain an HLH dimerization domain but lack the basic region that is required for DNA binding and form heterodimers with E proteins, antagonizing the DNA binding activity of E proteins and functioning as negative regulators of E proteins (16–18). Id proteins include Id1, Id2, Id3 and Id4, and hematopoietic cells primarily express Id2 and Id3. It is well established that the E and Id protein axis (the E-Id axis) regulates developmental trajectories of adaptive lymphocytes (19–21). The E2A gene encodes the E12 and E47 proteins, and E47 primarily regulates B cell lineage commitment, along with Ebf1, Pax5, and Foxo1 (22, 23). For T cell lineage commitment, E2A acts in pro-T cells along with HEB to establish a T cell-specific gene expression program and to suppress ILC development (24–28). HEB is also required for iNKT cell development from DP cells (29), and HEB and E2A play an important role in positive selection of DP cells (30). In contrast, *Id3* is upregulated by pre-TCR and γδ TCR signaling through ERK-MAPK, Egr1, and NFAT and plays a central role in αβ/γδ T cell fate and maturation (31–33). Furthermore, a recent report revealed the importance of the Notch-E2A-Tcf1 axis in αβ versus γδT cell lineage bifurcation and γδT cell function (34). In addition, E2-2 is critically required for interferon-producing plasmacytoid DC (pDC) development, while Id2 regulates antigen-presenting classical DC (cDC) development by neutralizing E2-2 activity (35–37). Furthermore, Id2 is well known as a critical regulator of the development of all ILC subsets, including ILC1-3s, NK cells, and lymphoid tissue inducer (LTi) cells (38, 39).

Many reviews describing the role of the E-Id axis have focused on the lineage commitment of T and B cells and DCs and on development of conventional T cells, NK cells, γδT cells, and iNKT cells. In this review we focus on the roles of the E-Id axis in T cell lineage commitment, including adaptive versus innate lymphoid cells, and during Treg cell differentiation.

## Adaptive Versus Innate Lymphoid Cells

ILCs are a family of lymphocytes that do not have diversified antigen recognition receptors, such as Ig and TCR, and that primarily reside in various tissues and respond to infection, injury and damage (40). ILCs modulate immune responses and contribute to the maintenance of tissue homeostasis by sustaining appropriate immune responses at mucosal barriers and by enhancing immune responses through secretion of inflammatory cytokines. Functional similarities regulated by a common set of specific TFs may suggest that ILCs are the innate counterparts of T cells. ILCs can be segregated into distinct classes according to effector cytokine secretion and expression of specific TFs. ILC1s, including NK cells, are characterized by secretion of interferon-γ (IFN- γ) and expression of the specific TF T-bet. ILC2s express the TF Gata3 and Th2 cytokines (interleukin-4 (IL-4), IL-5, and IL-13). ILC3s, including LTi-like cells, express Rorγt and secrete IL-17/IL-22 and lymphotoxin (40, 41). Therefore, ILC1s, ILC2s, and ILC3s are counterparts of CD4 helper T_H_1, T_H_2, and T_H_17 cells, respectively, while NK cells mirror CD8 cytotoxic T cells. As well as adaptive T and B lymphocytes, ILCs develop from common lymphoid progenitors (CLPs), and lineage commitment into ILCs is regulated by sequential expression of an ensemble of TFs, including Nfil3, Tox, Id2, Tcf1, and Gata3 (42–48). In addition, PLZF in ILC precursors (ILCp), Bcl11b and Rorα in ILC2s, and Runx3 in ILC1s/3s are required for this process (49–52). In particular, it is well known that Gata3, Tcf1, and Bcl11b are also required for early T cell development (3, 53). These observations clearly show close similarities between ILC and T cell lineages not only in effector function but also in their development, and a combination of these shared TFs determines effector functions in each lineage of ILCs after passing the developmental bifurcation of adaptive and innate lymphoid lineage commitment. However, how these shared TFs play their distinct roles in early T cell and ILC development remains to be clarified. Therefore, it is important to understand what events result in the differences between T cells and ILCs during their development.

**Figure 1:**
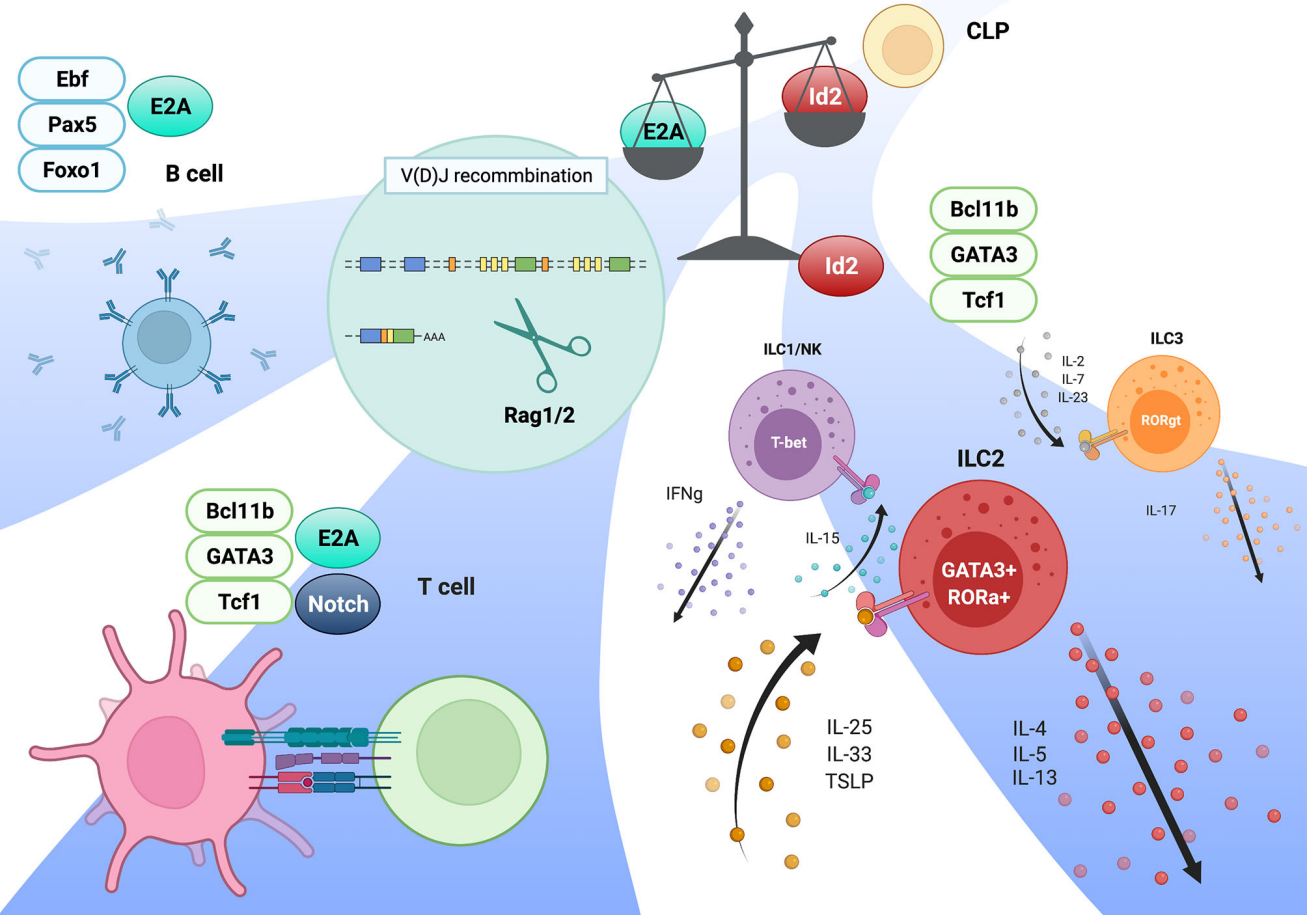
Model of adaptive and innate lymphocytes lineages mediated by the E-Id axis. The magnitude of E protein transcriptional activity determines the lineage commitments of adaptive versus innate lymphocytes. Following this process, an ensemble of TFs specific for each lineages validates lineage-specific gene expression program, along with E proteins in T and B cells. This figure was created with BioRender.com.

ILCs are derived from CLPs in the fetal liver (FL) and adult bone marrow (BM), and differentiate into functional mature ILCs in the resident tissues, while CD4 helper T cells and CD8 cytotoxic T cells mature in the thymus. The frequencies of ILCs, including mature Id2- and Gata3-expressing ILC2s and PLZF-expressing ILCps, are considerably low in the thymus of normal adult mice (54), because the majority of thymocytes in adult thymus are developing T cells. Consistent with a report that Rag1/2-mediated TCR recombination is dispensable for ILC development (55, 56), we and another group observed both the absence of D-J and V-DJ recombination of the TCRβ gene in ILC2s from wild-type lung tissue and aberrant ILC2s in the thymus from E2A/HEB-deficient mice (28, 57). According to these observations, the cell fate of the T versus ILC lineage must be principally determined by the thymic microenvironment. Notch signaling is one of the most likely external or environmental factors that distinguish T cells from the ILC lineage. In the absence of DL4 in thymic stromal cells, aberrant ILC2s are observed in the thymus, and constitutive Notch signaling completely blocks the ILC lineage *in vivo*. However, the proliferation of committed ILC precursors require mild to moderate Notch signaling, and short exposure to a Notch ligand combined with a high amount of IL-7 in CLPs leads to ILC2 generation *in vitro* (6, 58). Interestingly, recent studies have revealed an unexpectedly close relationship between T cells and ILCs (57, 59). Specifically, ILCps in BM express high levels of TCRβ constant region transcripts, and a proportion of tissue-resident ILC2s have undergone TCRγ gene recombination and express high levels of mRNAs of TCRβ and TCRγ4 constant regions (Cβ1/2 and Cγ4); however, the frequency of these TCRγ gene recombination is low, compared to that in γδT cells, and the recombination in these cells are nonfunctional (28, 57). Consistent with this observation, a high level of mRNA expression and broad chromatin accessibility in the TCRβ constant region with little or no expression of any TCR Vβ region in E2A/HEB-deficient ETPs, which tend toward an aberrant ILC lineage, were detected (28). According to these observations, T precursor cells that fail to properly undergo TCR recombination, especially TCRγ/δ recombination, may be able to convert their cell lineage into ILCs (56, 57). However, the numbers of mature ILC2s and PLZF-expressing ILCps in *Rag2*-deficient thymuses remain low; this phenomenon cannot explain why TCRγ/δ genes, but not TCRβ D-J gene, recombination are observed in ILCs, although TCRβ D-J and TCRγ/δ recombination occurs concurrently in the DN2 stage (28, 57). In contrast to these reports, the Sun group demonstrated that ILC2s in the thymus and lug from wild-type and *E2A/HEB* deletion (plck-Cre) mice, but not from *Id1-*transgenic (*Id1-Tg*) mice, exhibited TCRβ D-J and V-DJ gene recombination, which are detected by Southern blotting, and estimated that around 10% of ILC2s performed these recombination (60). In this report, even committed DN3 cells have a potential to differentiate into ILC2s *in vitro*, suggesting the lineage conversion of T cells to ILCs (60). Although these phenomena remain puzzling, T cells and ILCs are very close counterparts, and Rag1/2-mediated TCRβ recombination and its expression seem to be functional hallmarks of physiological T cell lineage commitment *in vivo*. A recent study provided an important clue regarding the mystery of the checkpoint for T cells and ILC2s in the thymus (61). During embryogenesis, functional ILC2s differentiate from ETPs in the fetal thymus, and these ILC2s preferentially migrate to mucosal tissues and reside for a long period. In this time-restricted thymic ILC2 development, specific TF RORα is the key factor that promotes ILC2 development and simultaneously suppresses the T cell lineage program by inducing *Id2* expression, leading to E2A function antagonism (61, 62). This study demonstrated that ILC2 development in E2A/HEB-deficient mice does not represent simple aberrant ILC development and instead may be an implication of the physiological embryonic thymocyte development toward the ILC2 lineage. Although *Id2* expression is a critical regulator of the ILC lineage, *Id2* deletion in E2A/HEB deficiency leads to thymic ILC development as well as E2A/HEB deficiency, and transient *Id2* expression induced by doxycycline can induce aberrant ILC2 development in adult thymus. Thus, T cell and ILC lineages may simply depend on the magnitude of E protein activity, and Id2 may function as a lineage switch for ILCs (28). Therefore, we conclude that after the enhancer repertoire associated with each lineage regulated by the E-Id axis is established, an ensemble of shared TFs, such as Tcf1, Bcl11b, and Gata3, instructs the lineage-specific gene expression programs in both T cells and ILCs (**Figure 1**). Indeed, Bcl11b binds to different sites in a lineage-specific manner associated with cell type-specific protein complexes (63). Interestingly, some members of these factors are dynamically recruited to the regulatory regions not only in a lineage-specific manner but also in a developmental stage-specific manner (64).

However, it remains unclear whether the loss of E protein activity in ETPs induces only ILC lineage commitment or also leads to the expansion of ILC precursors or mature ILCs. Since Id2 is continuously expressed at high levels after ILC lineage commitment, the magnitude of E protein activity may control not only the ILC versus T lineage commitment but also the expansion or activation of ILCs after the commitment, which is antagonized by Id2. Because E2A functions both as an initiator upon T cell lineage commitment and as a gatekeeper at β-selection (65), the loss of E protein activity in ILCs may play a role in the activation or expansion of ILCs.

How is the E-Id axis regulated? While E2A and HEB mRNA expression levels are relatively consistent throughout the thymocyte development (ImmGen data; https://www.immgen.org/), the E2A protein level is high in ETPs and is the highest in DN2 cells; this level is downregulated in resting DP cells, as revealed by E2A-GFP fusion knock-in mouse analysis, indicating the presence of posttranslational regulation of the E2A protein (66–68). On the other hand, *Id3* is upregulated by TCR signaling, including pre- and γδ-TCR, during thymocyte development and remains at a high level in peripheral naïve T and Treg cells (32, 67, 69). In peripheral T cells, TCR stimulation induces E2A protein expression, which is required for rapid memory-precursor formation of CD8 T cells, while Id2 and 3 function as regulators of CD8 T cell responses (70). Surprisingly, differential *Id2* and *Id3* expression in CD4 T cells during viral infection regulates T_H_1 or T_FH_ cell development, respectively (71). During ILC lineage commitment, *Id2* is initially upregulated in PLZF-expressing ILC precursors in which E2A protein is already downregulated, and this induction of *Id2* expression is associated with the IL7R expression level, suggesting the involvement of cytokine signaling in *Id2* expression (28). Consistently, the cis-regulatory element of the *Id2* gene, which expresses the long noncoding RNA Rroid, controls ILC1 function by regulating Stat5 deposition at the *Id2* promoter region; however, this locus is dispensable for *Id2* expression in other ILCs (72). Therefore, *Id2* expression in ILC lineages, which is probably mediated by cytokine signaling, is required not only for ILC lineage commitment but also for ILC maintenance.

## *Rag1* and *Rag2* Gene Expression Mediated by E Proteins

As we discussed in the introduction, *Rag1/2* gene expression discriminates between adaptive and innate lymphoid lineages. This indicates that TFs responsible for *Rag1/2* expression are critical regulators of T and B lineage commitment (73). There are two waves of Rag1/2 expression during T and B cell development (74). The first wave of *Rag1/2* expression is required for the assembly of IgH and TCRβ genes in pro-B and pro-T cells, respectively. After the selection of pre-TCR (TCRβ) or pre-BCR (IgH), Rag1 expression is transiently downregulated during the transition from the progenitors to precursors. In the precursor stage, Rag1/2 are re-expressed for IgL and TCRα gene recombination. Following the positive and negative selection of the TCR or BCR, the *Rag1/2* genes are suppressed in mature naïve T and B cells and are never expressed for further recombination of the *TCR* and *Ig* genes. During these developmental processes, *Rag1/2* gene expression is tightly regulated, and other types of immune cells never express the *Rag1/2* genes. However, the molecular mechanisms of *Rag1/2* gene expression remained to be determined. Both *in vivo* and *in vitro* studies have attempted to define the enhancer regions and TFs responsible for *Rag1/2* expression (75). Both T and B progenitor/precursor cells express *Rag1/2* and require distinct enhancers of these genes. The deletion of *Erag* (*Enhancer of Rag*), which is located at 23 kb upstream of the *Rag2* promoter, resulted in a significant reduction in *Rag1/2* expression and partial developmental defects during B cell development, without affecting thymocyte development (76). A study has reported that this *Erag* region is positively regulated by Foxo1 and negatively regulated by Gfi1b, Ebf1, and c-Myb (77–80). In contrast, an anti-silencer element (*ASE*), which is 8 kb in length and located 73 kb upstream of the *Rag2* promoter, is required for *Rag1/2* gene expression in DN3 and DP cells but not in developing B cells (81). In ChIP-seq data, most of T cell TFs includng E2A, Bcl111b, Tcf1, Gata3, Runx1, and Ikaros bound to ASE regions, while B cell TFs such as E2A, Pax5, and Irf4, but not Ebf1, bound to Erag region (82, 84).

The Krangel group demonstrated that the chromatin organizer mediates the interaction between *ASE* and *Rag1/2* promoters to promote optimal expression of the *Rag1/2* genes in DP cells and suggested that the *ASE* and *Rag1* promoter regions function as a chromatin hub (82). Furthermore, this group proved that Gata3 and E2A regulate the *ASE* region, and *Rag1* promoter activity relies on Runx1 and E2A binding in the VL3-3M2DP thymocyte cell line (83). A study also identified T or B cell-specific enhancer elements that drive *Rag1/2* expression using the E2A ChIP-seq and ATAC-seq data from pro-T and pro-B cells to clarify the regulatory mechanisms of adaptive versus innate lineage choice. Two B cell-specific enhancers (*Rag* B cell enhancer 1 and 2; *R1B* (5 kb upstream of the *Rag1* promoter) and *R2B* (partially overlapping with *Erag*)) and one T cell-specific enhancer (*Rag*-T cell enhancer (*R-TEn*)) were identified (84). A common E2A-binding region near the *Rag1* promoter (*R1pro*), which is shared between T and B cells, was also identified. *R1B/R2B* and *R-TEn* uniquely bind to the *Rag1/Rag2* promoter regions and form distinct chromatin structures in developing T and B cells, respectively. Deletion of both *R1B* and *R2B* in mice resulted in a severe developmental block at the pro-B stage, but not in T-cell development, resulting from drastic impairments in Rag-mediated IgH gene recombination, whereas single deletion of either *R1B* or *R2B* resulted in mild-to-moderate defects in B cell development that also occurred in *Erag* deletion mice (76, 84). This finding suggests enhancer redundancy in *Rag1/2* expression in B cells. In contrast, *R-TEn* deletion resulted in severe developmental defects in β-selection of DN3 cells and positive selection of DP cells without affecting B cell development (84). These results raised the question of what TF regulates these *Rag* gene enhancer regions.

E2A is especially notable among TFs responsible for adaptive lymphocyte development because *Rag1/2* gene expression was significantly reduced in E2A-deficient lymphoid-primed multipotent progenitors (LMPPs) and T progenitor cells (28, 85, 86). A mutation of the E-box motifs in the *R-TEn* (*R-TEn-E-box-mutant*), which blocks E-protein binding without affecting the recruitment of other TFs to this enhancer, directly proves that the E2A/E protein regulates this enhancer. *R-TEn-E-box-mutant* mice showed developmental defects in β-selection and positive selection, resulting from a severe reduction in *Rag1/2* gene expression in DN3 and DP cells. Furthermore, genome structures, chromatin accessibility, histone H3 lysine K27 acetylation (H3K27ac), and cohesin recruitment were completely lost only at the *Rag* gene locus, indicating that the E2A/E protein binding to the enhancer region induces and promotes cell type-specific superenhancer (SE) formation (84). How does the E2A/E protein induce SE formation? bHLH TFs, such as E2A, were reported to interact with the histone acetyltransferase (HAT) CBP/P300 and SAGA proteins through the PECT motif within the activation domain 1 (AD1) of the E protein and recruit these coactivators to enhancer regions, thus inducing and promoting H3K27 acetylation (87–91). Active enhancers are accompanied by high levels of H3K27ac, CBP/P300, chromatin remodeler Brg1, and RNA polymerase II (PolII) to facilitate the recruitment of cohesin-loader NIPBL and the cohesin complex, which induce large-scale structural changes of the chromatin and may switch the locus from transcriptionally repressive (B) to permissive (A) compartments (92, 93). Simultaneously, E2A and other specific TFs also recruit the ten-eleven translocation (TET) family proteins to the enhancers to remove DNA methylation of the CpG islands in enhancers, which is associated with the SE function in developing and activated B cells (94, 95). SEs regulate certain genes that play characteristic roles in cell type-specific functions, thereby establishing cell identity (96, 97). Because the properties of SEs are based on highly cooperative interactions between cell type-specific TFs, transcriptional mediators, and RNA PolII and due to vulnerability to a perturbation of the key protein components (98), E2A functions in adaptive lymphocyte-specific enhancer regions as a pioneer and maintainer. Additionally, E2A plays an essential role in *Rag1* expression *in vivo* through the regulation of the promoter activity. Surprisingly, E-box motif mutations in the *Rag1*-promoter region (*R1pro-E-box-mutant*) alone in mice are sufficient to inhibit the *Rag1* gene expression, which leads to the developmental arrest at both the T and B cell progenitor stages, similar to those in *Rag1-*deficient mice. However, *Rag2* expression and enhancer regions (*R-TEn* and *R1B/R2B*) are not affected in *R1pro-E-box-mutant* DN3 and pro-B cells (84). This result indicates that both cell type-specific enhancer and promoter regions independently rely on the recruitment of the E2A/E protein and that E protein-mediated interactions between enhancer and promoter regions determine adaptive lymphocyte-specific expression of the *Rag* gene. We summarised these regulatory regions in **Table 1**.

**Table 1:** Description of Rag gene enhancer regions.

cis-regulatory element	Length/open	TF bindings by ChIP-seq data	Defects in deletion or mutant mouse	Rag1/Rag2 expression	Ref paper
anti-silence element (ASE)	8 kb		defects in thymocyte development (DN3, DP)	Rag1/2; down in DP cells	(79)
Enhancer of Rag (Erag)	1.7 kbp	E2A, Ets1, Ikaros	moderate defect in B cell development	Rag1/2; down in developing B cells	(74)
Rag-B cell enhancer 1 (R1B)	<1 kb	E2A, Ikaros, Irf4	mild defect in B cell development	moderate reduction of Rag1/2 expression	(80)
Rag-B cell enhancer 2 (R2B)	2 kb (partially overlapped with Erag)	E2A, Pax5, Ets1, Ikaros	moderate defect in B cell development	moderate reduction of Rag1/2 expression	(80)
R1B/R2B	R1B/R2B double deletion		developmental arrest at pro-B stage	drastic reduction of Rag1/2 expression in pro-B cells, but not in T cell	(80)
Rag-T cell enhancer (R-TEn)	2 kb (included in ASE)	Satb1, E2A, Ikaros, Bcl11b, Tcf1, Runx1, Gata3	defects in thymocyte development (DN3, DP)	Rag1/2; down in DN3a and DP cells	(80)
R-TEn peak 1	open in DN3/DP		defects in thymocyte development (DN3, DP)	Rag1/2; down in DN3a and DP cells	(80)
R-TEn peak 2	open in DP		no defect	normal	(80)
R-TEn peak1 E-box mutant	blocking E-protein binding to R-TEn		defects in thymocyte development (DN3, DP)	Rag1/2; down in DN3a and DP cells	(80)
Rag1 promoter E-box mutant	blocking E-protein binding to Rag1 promoter		developmental arrest at pro-B and DN3 stages	defects in Rag1, but not Rag2, expression in DN3a and pro-B cells	(80)

**Figure 2:**
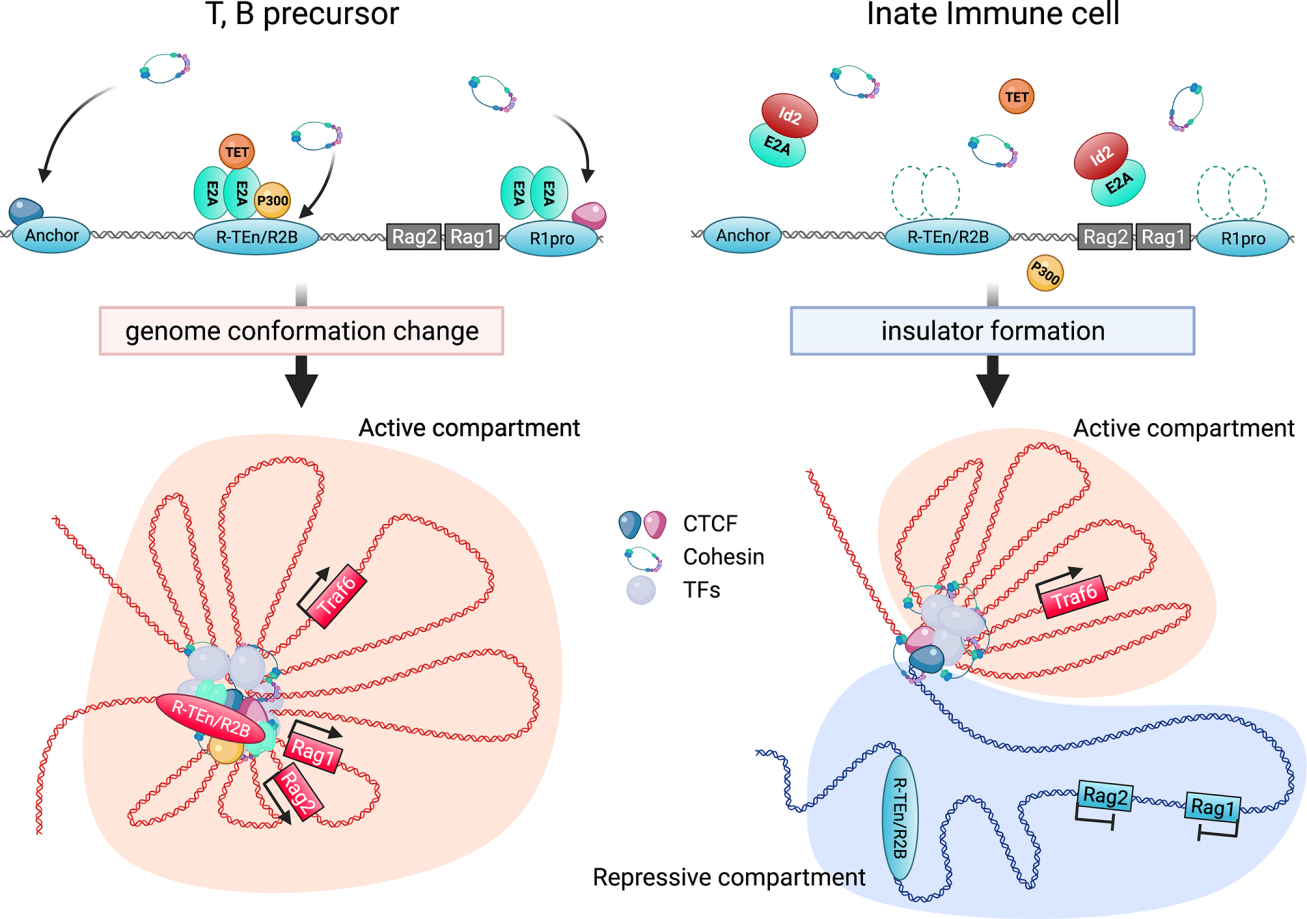
Regulation of *Rag* gene locus by E2A and cis-regulatory elements. E2A binding to the specific enhancer (*R-TEn* and *R2B*) and *R1pro* regions induces the genome conformation changes to form adaptive lymphocyte-specific SE through the recruitment of P300, TET, and NIPBL-cohesin complex (left; developing T and B cells). In contrast, Id2 prevents E2A/E proteins from binding to these regulatory regions, leading to the insulator formation to sequester the *Rag* genes in repressive chromatin compartment in innate immune cells (right; macrophage etc). This figure was created with BioRender.com.

Overall, the binding of the E2A/E proteins to the E-box motifs in the cell type-specific cis-regulatory regions induces the recruitment of P300, other transcription mediators, the NIPBL/cohesin-complex, and chromatin organizers to orchestrate 3D structural changes of the genomes to initiate and maintain cell type-specific gene expression. In contrast, high expression levels of Id2 prevents *Rag* gene SE formation by antagonizing the E2A activity, and the *Rag* gene is sequestered in repressive chromatin (B) compartment (**Figure 2**). Curiously, sequence similarities of T and B cell-specific *Rag* gene enhanceres are conserved among mammals, birds and reptiles, but not in amphibians and fish. In addition, these conserved enhancer regions have been shown to harbor the E-box motifs conserved among these species (84). Thus, we propose that terrestrial animals evolutionarily acquired the gene regulatory mechanism mediated by the E proteins as enhancers to achieve higher *Rag* gene expression, which enables a diverse range of *TCR* and *Ig* gene recombination to protect against a wide range of the pathogens (99).

## Treg Cells and the Role of the E-Id Axis

**Figure 3:**
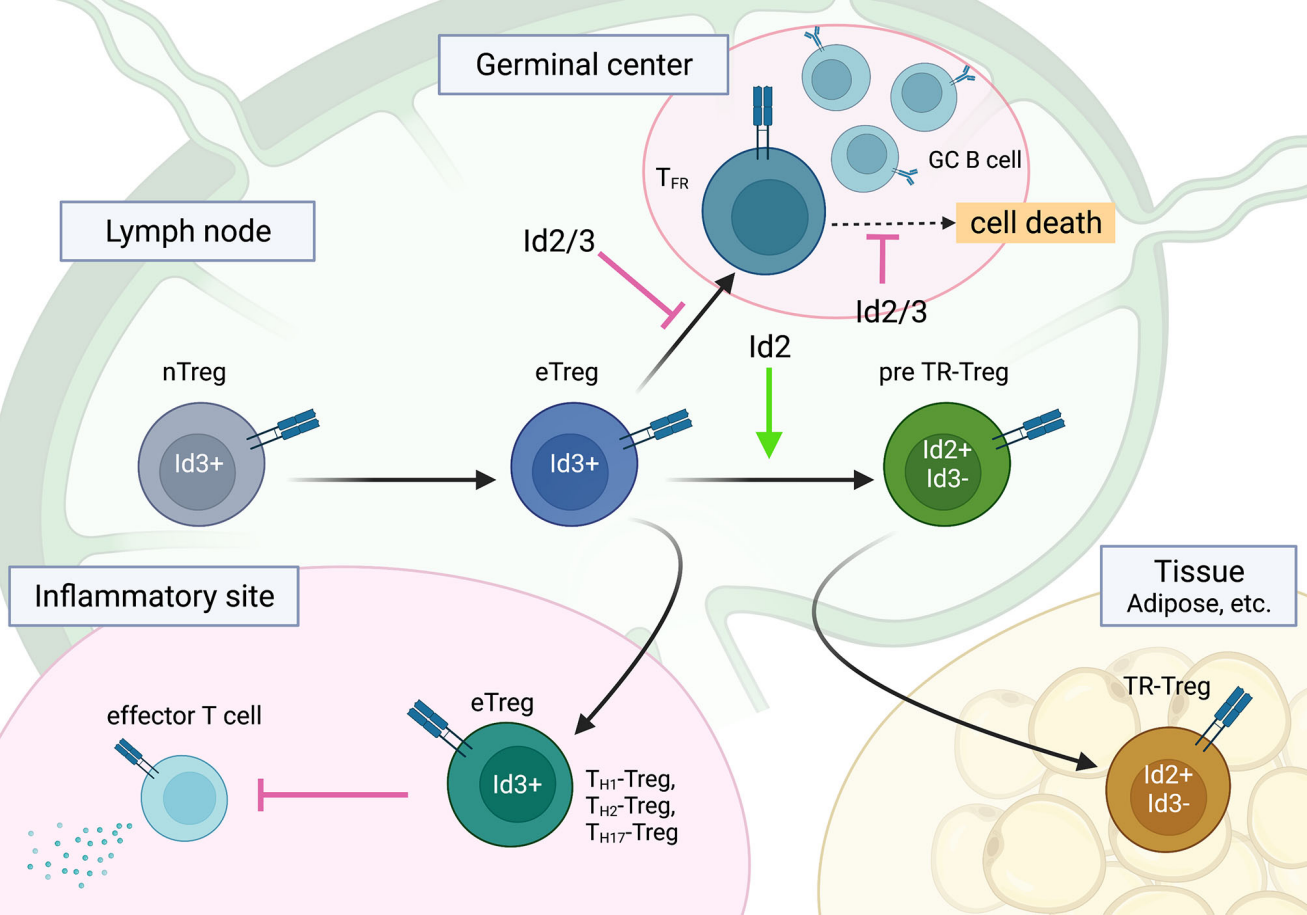
The roles of Id2 and Id3 in Treg cell differentiation into subsets of effector Treg cells. Id2 and Id3 enforce the naïve state of Treg cells, especially in T_FR_ cells. A regulatory switch of Id3 to Id2 plays a role in TR-Treg cell differentiation and function. This figure was created with BioRender.com.

E and Id proteins play a central role in effector/memory and tissue-resident cytotoxic CD8 T cell differentiation and the activation of helper CD4 T cells, including T_H_1 and follicular helper T (T_FH_) cells (67, 71, 100–105). However, to our knowledge, no review papers have addressed the role of the E-Id axis in Treg cells. In this section, we focus on the roles of Id and E proteins in Treg cells. Treg cells play a central role in the maintenance of immune homeostasis by suppressing autoimmunity and excessive inflammatory responses and by tissue repair after inflammation. Naturally occurring Treg cells differentiate in the thymus (natural Treg (nTreg) or thymic Treg (tTreg) cells), which constitutively express TF Foxp3, while a population of Foxp3-expressing Treg cells develops from naïve CD4 T cells in the periphery (peripheral Treg (pTreg) cells) (106). In addition, naïve CD4 T cells can develop into Foxp3-expressing Treg cells *in vitro* by TCR stimulation in the presence of TGF-β plus IL-2 (induced Treg (iTreg) cells) (107). Treg cells show functional heterogeneity to regulate a variety of immune responses, and each subset of Treg cells has a specialized gene expression program. As well as conventional CD4 T cells, Treg cells differentiate into effector subsets, named effector Treg (eTreg) cells, accompanied by Blimp1 and Irf4 TFs, and express unique migratory chemokine receptors to home to the site of inflammation and higher suppressive molecules such as IL-10 and CTLA-4 to control tissue inflammation (108–110). For instance, T_H_1-Treg cells express CXCR3, which is mediated by T-bet, to migrate into T_H_1 inflammatory sites (111). In addition, follicular regulatory T (T_FR_) cells, a specialized subset of Treg cells, regulate T_FH_ cell function and germinal center B-cell responses for the humoral immunity (112–114). More recently, specialized subsets of Treg cells in nonlymphoid tissues, such as adipose tissue, muscle tissue, lung tissue, and the central nervous system, have been shown to play an important role in tissue homeostasis and regenerative functions, and amphiregulin and Notch ligand Jagged1 from Treg cells contribute to tissue regeneration (115–118). This subset of Treg cells is often referred to as tissue-resident Treg (TR-Treg) cells. They are derived from effector Treg cells, which in turn are instructed by TF Batf (119) (**Figure 3**).

There are many previous studies about the role of the E-Id axis in Treg cell development and activation. The expression of *Id3* is high in naïve Treg cells and low in ICOS^+^ effector Treg cells, and TCR stimulation in Treg cells downregulates *Id3*. In contrast, *Id2* levels are low in naïve Treg cells, and TCR stimulation induces the upregulation of *Id2 in vitro* (120, 121). It has been reported that *E2A/HEB* and *Id3* are involved in the development of tTreg cell and iTreg cells; drastically increased tTreg cells were observed in a study of *E2A/HEB*-deficient thymus, while decreased tTreg cells were detected in a study of *Id3* ^–/–^ thymus (122, 123). In addition, blocking the E protein by *Id1* overexpression in mice resulted in an increased frequency and number of tTreg cells due to the expansion of thymic Treg cells, while Foxp3 mRNA induced by TCR stimulation was significantly lower in naïve *Id1-*Tg CD4 T cells (124). However, the deletion of *E2A* and *HEB* in early stages blocks T cell lineage commitment, and their deletion in DP cells bypasses the TCR-mediated positive selection of DP cells, leading to the CD8SP stage accompanied by severe impairment of the CD4SP lineage (28, 125). In addition, *Id3* is required for MHC-restricted positive selection of DP cells (126). The combined loss of *Id2* and *Id3* results in blockage of the transition from CD69^+^TCRβ^lo or –^ DP to fully TCR-selected CD69^+^TCRβ^hi^ DP cells at a young age; however, PLZF-expressing innate T_FH_ cells expand with limited TCR repertoires and occupy the CD4SP population in adults, suggesting that in the absence of *Id2* and *Id3*, conventional CD4 T cell development is severely affected (102). Therefore, it remains unclear whether changes in tTreg populations in these gene-deficient mice are reflected by the severely impaired CD4SP population and reduced strength of TCR signaling or whether E2A/HEB and Id3 are actually involved in the induction of Foxp3 expression or tTreg cell development. Furthermore, since Id3 enforces naïve T cell fate by antagonizing E2A activity and *Id3*-deficient CD4SP or CD8SP cells readily differentiate into IFN-γ-producing effector T cells,T_FH_ cells (CD4SP), or innate-like CD8 T cells in the thymus (67, 127), attenuated iTreg cell development in *Id3* ^–/–^ mice is more likely the result of fewer naïve CD4 T cells in the periphery. However, from the result that the deletion of *E2A/HEB* led to increased iTreg development *in vitro*, E protein activity is thought to be involved in iTreg cell development (123). It was reported that E47 indirectly regulates Foxp3 expression through the regulation of Spi-B and SOCS3 in *Id3*-deficient Treg cells and that *Foxp3* mRNA in *Id2/Id3-*deficient Treg cells is comparable to that in control Treg cells, indicating that E2A does not regulate *Foxp3* gene expression (120, 128). In line with this, E2A occupancy around the *Foxp3* gene locus, by ChIP-seq analysis, was not detected in *Id2/Id3-*deficient DP cells (129).

Although the role of Id and E proteins in tTreg development is unclear, the E-Id axis plays an important role in Treg cell function. Indeed, Treg-specific deletion of *Id2* and *Id3* using Foxp3-Cre in mice leads to fatal inflammatory disease, which is characterized by spontaneous T_H_2 inflammation in the lung, skin, and esophagus, similar to human atopic diseases such as bronchial asthma, atopic dermatitis, and eosinophilic esophagitis (120). *Id2/Id3* depletion in Treg cells induces CXCR5, which is a direct target of the E2A-Id3 axis in T_FR_ and T_FH_ cell development and preferentially migrates to B-cell follicles. However, *Id2/Id3-*deficiency in Treg cells has been shown to result in compromised maintenance of Treg cells mediated by TCR stimulation *in vitro*. This result suggests that Id proteins function as gatekeepers for eTreg and T_FR_ cells as well as CD4 T cells and control the maintenance of Treg cells. Although *Id2* and *Id3* compensate for each other in single KO Treg cells, Id2 and Id3 have distinct roles in Treg cell function. According to *Id3* expression with CD62L and CD44, the Campbell group demonstrated stepwise developmental stages toward TR-Treg cells; *Id3* was highly expressed in central naïve Treg cells and effector Treg cells, whereas ICOS^hi^ *Id3* ^lo^ TR-Treg precursor cells expressed *Id2*, suggesting a regulatory switch from Id3 to Id2 in Treg cells (121, 130). This seems to be similar to tissue resident effector/memory CD8 T cells (100, 105). Interestingly, consistent with the Id switch in Treg cells, a loss of *Id2* expression in Treg cells results in decreased expression of TR-Treg cell-related functional molecules and leads to increased cell death of Treg cells, suggesting an Id2-dependent TR-Treg cell-specific program (131). Curiously, Treg cells lacking E2A and HEB exhibit effector phenotypes and increased stability, suggesting the linkage of E protein and TCR signaling in the gene signature of effector Treg cell development (132). In contrast, ectopic *Id2* expression in Treg cells in mice enhance Treg cell plasticity and lead to a reduction in Treg cells (133). Taken together, although the underlying molecular mechanism remains to be determined, it now seems apparent that the E-Id axis orchestrates Treg cell differentiation toward the fate of T_FR_, eTreg and TR-Treg cells and dictates function and plasticity in lymphoid and nonlymphoid tissues (**Figure 3**).

## Conclusion

The E-Id transcriptional axis plays an important role in T/B cell lineage commitment, discrimination between T cells and ILCs, including *Rag* gene expression, and T/Treg cell function. However, it remains to be investigated how the E-Id axis orchestrates cell type-specific enhancer activities in conjunction with other TFs associated with T cell activation and TCR signaling. Future experiments are warranted to explore the role of the E-Id axis in T and B cell activation under the inflammatory conditions. These findings may have implications for health and immunological disorders.

## Author Contributions

RH, KM and MM wrote the manuscript and figures. All authors contributed to the article and approved the submitted version.

## Funding

This work was funded by the KAKENHI (Grants-in-Aid for Scientific Research) from the MEXT of Japan (19H03487 for MM), the Mochida Memorial Foundation, the Takeda Science Foundation, the FUJIWARA Memorial Foundation (MM).

## Conflict of Interest

The authors declare that the research was conducted in the absence of any commercial or financial relationships that could be construed as a potential conflict of interest.

## Publisher’s Note

All claims expressed in this article are solely those of the authors and do not necessarily represent those of their affiliated organizations, or those of the publisher, the editors and the reviewers. Any product that may be evaluated in this article, or claim that may be made by its manufacturer, is not guaranteed or endorsed by the publisher.
